# Molecular diversity and predictability of *Vibrio parahaemolyticus* along the Georgian coastal zone of the Black Sea

**DOI:** 10.3389/fmicb.2014.00045

**Published:** 2014-02-10

**Authors:** Bradd J. Haley, Tamar Kokashvili, Ana Tskshvediani, Nino Janelidze, Nino Mitaishvili, Christopher J. Grim, Guillaume Constantin de Magny, Arlene J. Chen, Elisa Taviani, Tamar Eliashvili, Marina Tediashvili, Chris A. Whitehouse, Rita R. Colwell, Anwar Huq

**Affiliations:** ^1^Maryland Pathogen Research Institute, University of MarylandCollege Park, MD, USA; ^2^George Eliava Institute of Bacteriophages, Microbiology and VirologyTbilisi, Georgia; ^3^University of Maryland Institute for Advanced Computer Sciences, University of MarylandCollege Park, MD, USA; ^4^MIVEGEC UMR 5290 IRD-CNRS-UM1&2, IRD de MontpellierMontpellier, France; ^5^U.S. Army Medical Research Institute of Infectious DiseasesFort Detrick, MD, USA; ^6^Bloomberg School of Public Health, Johns Hopkins UniversityBaltimore, MD, USA; ^7^CosmosID™College Park, MD, USA; ^8^School of Public Health, Maryland Institute for Applied Environmental Health, University of MarylandCollege Park, MD, USA

**Keywords:** *Vibrio parahaemolyticus*, predictive modeling, *Vibrionaceae*, Black Sea, aquatic microbiology

## Abstract

*Vibrio parahaemolyticus* is a leading cause of seafood-related gastroenteritis and is also an autochthonous member of marine and estuarine environments worldwide. One-hundred seventy strains of *V*. *parahaemolyticus* were isolated from water and plankton samples collected along the Georgian coast of the Black Sea during 28 months of sample collection. All isolated strains were tested for presence of *tlh*, *trh*, and *tdh*. A subset of strains were serotyped and tested for additional factors and markers of pandemicity. Twenty-six serotypes, five of which are clinically relevant, were identified. Although all 170 isolates were negative for *tdh*, *trh*, and the Kanagawa Phenomenon, 7 possessed the GS-PCR sequence and 27 the 850 bp sequence of *V. parahaemolyticus* pandemic strains. The *V. parahaemolyticus* population in the Black Sea was estimated to be genomically heterogeneous by rep-PCR and the serodiversity observed did not correlate with rep-PCR genomic diversity. Statistical modeling was used to predict presence of *V. parahaemolyticus* as a function of water temperature, with strongest concordance observed for Green Cape site samples (Percent of total variance = 70, *P* < 0.001). Results demonstrate a diverse population of *V*. *parahaemolyticus* in the Black Sea, some of which carry pandemic markers, with increased water temperature correlated to an increase in abundance of *V. parahaemolyticus*.

## Introduction

*Vibrio parahaemolyticus*, a halophilic bacterium, is a causative agent of seafood-related gastroenteritis, wound infections, and septicemia and is known to occur in marine, estuarine, and brackish water environments globally with sporadic occurrence in fresh water (Sarkar et al., 1985; DePaola et al., 2000; Wong et al., 2000; Alam et al., 2009). In addition to notoriety as a causative agent of human infection, the organism is autochthonous to marine and brackish water ecosystems and, similar to other *Vibrio* spp., degrades chitin (Kaneko and Colwell, 1974; Kadokura et al., 2007). One of its main virulence factors, the type three secretion system-2 (TTSS2), plays an important role in preventing predation of its host by higher organisms, suggesting the virulence factors have evolved via environmental selection (Matz et al., 2011). Little work has been done on non-anthropocentric roles of this organism, but its ubiquity and association with animals demonstrate that its ecology extends beyond the human body.

The majority of clinical strains encode the thermostable direct hemolysin (TDH), within the *V. parahaemolyticus* pathogenicity island (Vp-PAI), one of the virulence factors responsible for enterotoxicity (Honda, 1993; Guang-Qing et al., 1995). However, some clinical isolates do not encode TDH, but other hemolysins instead, such as the TDH-related hemolysin (TRH), while all encode the thermolabile hemolysin (TLH). It has also been reported that two type three secretion systems (TTSS1 and TTSS2) are involved in *V. parahaemolyticus* pathogenicity (Bhattacharjee et al., 2006; Ono et al., 2006; Kodama et al., 2007; Matlawska-Wasowska et al., 2010). The TTSS1 found in all *V. parahaemolyticus* strains examined to date has been shown to translocate an effector protein (VP1686) into the cytosol of macrophages and induce DNA fragmentation and another effector protein (VP1680) has been shown to play a role in cytotoxicity in eukaryotic cells (Bhattacharjee et al., 2006; Ono et al., 2006). Interestingly, *V. parahaemolyticus* strains lacking TDH, TRH, and TTSS2 have frequently been isolated from patients not colonized by TDH-, TRH-, and TTSS2-positive strains, suggesting TTSS1 is also responsible for illness in humans (Suthienkul et al., 1995; Okuda et al., 1997; Vuddhakul et al., 2000; Laohaprertthisan et al., 2003; Cabanillas-Beltrán et al., 2006; Bhoopong et al., 2007; Meador et al., 2007; Serichantalergs et al., 2007; Chao et al., 2009, 2010; García et al., 2009; Harth et al., 2009).

*V*. *parahaemolyticus* has been frequently isolated from water samples collected from the Black Sea and sporadic cases of gastroenteritis caused by this bacterium and related vibrios have historically been reported in the Sea of Azov region (Libinzon et al., 1974, 1980, 1981; Shikulov et al., 1980; Clark et al., 1998; WHO, 2011). Further, human pathogenic vibrios are known to be endemic to the greater Caucasus (Narkevich et al., 1993; Gurbanov et al., 2011; Rashid et al., 2013) but the ecologies of these organisms are not well-elucidated in this region. The increasing global incidence of *V. parahaemolyticus* infections suggests it is important to fully understand the ecology of these regions in multiple locations so that public health assessments can be made more accurately (Baker-Austin et al., 2010). Members of the *Vibrionaceae* are known to have an intimate association with planktonic organisms and many studies have demonstrated the role of environmental conditions (namely water temperature and salinity) on the density of these organisms in water bodies. Generally, an increase in temperature of a water body is associated with an increase in *Vibrio* density (Turner et al., 2009; Oberbeckmann et al., 2012). To further understand the ecology of *V. parahaemolyticus* along the Georgian coast of the Black Sea we evaluated the presence of these organisms in water and plankton fractions over a 28 month period (June 2006 to October 2008) and modeled their presence in relation to environmental conditions (salinity, water temperature, pH, and dissolved oxygen). We further evaluated the molecular diversity and presence of virulence factors in a subset of *V. parahaemolyticus* isolates collected during this study.

## Materials and methods

**Figure 1:**
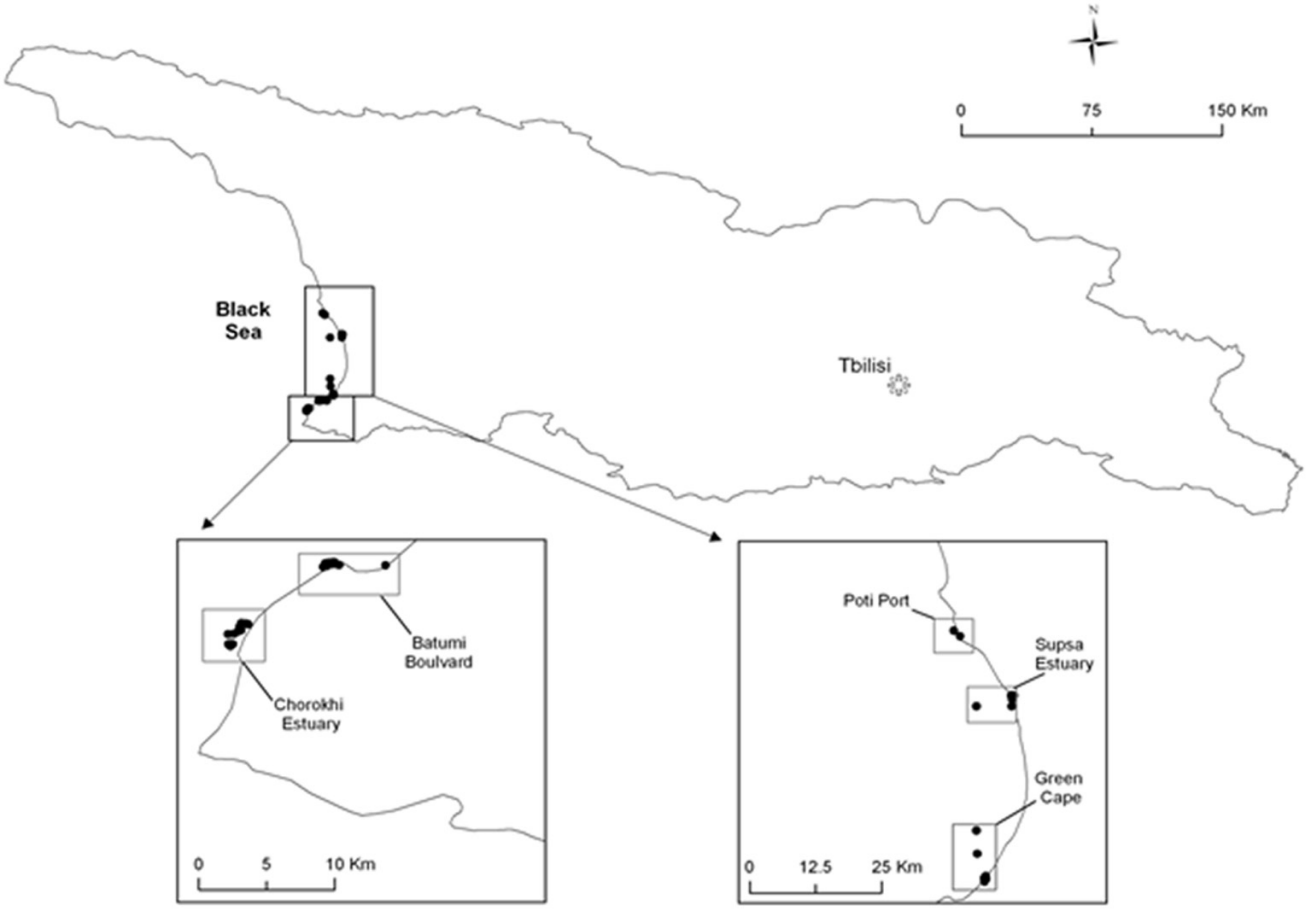
**Map showing locations of sampling sites along Black Sea**.

Water samples were collected monthly, except July to September when water was collected biweekly, from five stations on the coast of the Black Sea (Figure 1). One hundred liters of water were filtered through 200- and 64-μm plankton nets, to separate size fractions of plankton. Water temperature, salinity, pH, and dissolved oxygen were recorded at the time of sampling. The water fraction (100 ml) was filtered using a 0.45 μm nitrocellulose membrane, which was incubated in alkaline peptone water (APW) at 37°C for 24 h. An aliquot (1- to 5-ml) of each plankton fraction (64- and 200-μm) was also inoculated in APW and incubated at 37°C for 24 h. A 10 microliter loop of the enrichment cultures were streaked onto thiosulfate citrate bile salts (TCBS) agar plates, which were incubated overnight at 37°C. All colonies that appeared yellow to green at 24 h were considered presumptive *Vibrio* spp., picked with a sterile toothpick, and streaked to isolate colonies on Luria–Bertani (LB) agar. Presumptive *V*. *parahaemolyticus* colonies were confirmed by streaking onto CHROMagar™ *Vibrio* (mauve colonies) the latter were confirmed by PCR (presence of *tlh*, and *V. parahaemolyticus*-specific collagenase).

For molecular analyses, the following PCR primers were used; collagenase (Di Pinto et al., 2005), *tdh*, *trh*, and *tlh* (Bej et al., 1999), GS-PCR (Matsumoto et al., 2000), ORF8 (Nasu et al., 2000), Mtase (Wang et al., 2006), histone-like DNA-binding protein (HU-α ORF) (Williams et al., 2004), the 850 bp pandemic strain sequence (VPF2/VPR2) (Khan et al., 2002), VP1346 (*yop*) and VP1339 (*escC*) of TTSS2 (Chao et al., 2010), VP1680 (Whitaker et al., 2012) and VP1686 of TTSS1 (This study). Primer sequences for VP1686 were VP1686-F: TGCTTTTGTGATCGCTTTTG and VP1686-R: TGAAGGCAAACTCAGCATTG (*T*a = 56°C; amplicon size = 169 bp) and were designed *in silico* using *V. parahaemolyticus* RIMD2210633 (NC_004603.1/NC_004605.1). DNA (25.0 ng) was mixed with 2.5 mM of dNTP, 15 mM of PCR buffer, and 5 U μL^−1^ of Taq DNA polymerase, using 20 μm of appropriate primer for each analysis. Amplicons were visualized on 1.5% agarose gel stained with ethidium bromide and examined under a UV transilluminator.

To approximate the molecular diversity of the *V*. *parahaemolyticus* isolates, rep-PCR was executed on a randomly selected subset of strains following the methods of Chokesajjawatee et al. (2008). PCR products were separated on a 1% agarose gel in TAE buffer. The resulting fingerprint patterns were documented using the GelDoc-It™ Imaging System (Ultra-Violet Products, Upland, CA). Banding patterns were identified by visual observation and dendrograms were calculated by the unweighted pair-group method using average linkages (UPGMA). Serotyping was performed as follows. Strains were streaked on LB agar with 3% NaCl and incubated overnight at 37°C. One 10 μl loopful of growth was homogenized in 1 mL of saline solution (0.9% NaCl). This solution was divided into two 500 μl tubes, one of which was boiled for 2 h. Ten microliters of the boiled cell solution was then mixed with 10 μl of each O-antisera and 10 μl of the cell suspension that had not been boiled was mixed with 10 μl of K-antisera on a glass slide and agglutination visually determined (Denka Seiken Co., Niigata-ken, Japan). Distilled water was used as a negative control for serotyping assays. *V. parahaemolyticus* strain RIMD2210633 (KP positive; serotype O3:K6) for assays.

Predictive models of *V. parahaemolyticus* detection were determined by examining the relationship between presence/absence (response variable) and recorded environmental parameters (explanatory variables) at the time of sample collection. Environmental parameters were also evaluated as explanatory variables by determining the distance from optimality for each data point. This was performed by subtracting the median values of all parameters for those samples in which *V. parahaemolyticus* had been detected (optimal parameters) from all data points following the methods of Jacobs et al. (2010) and Banakar et al. (2011). The absolute values of differences were used as explanatory variables in binary logistic regression analysis. For all measures of association, *p*-values ≤ 0.05 were considered significant. Statistical analyses were conducted on R (http://www.r-project.org/) and SAS softwares (Cary, NC, USA).

## Results

### Detection of *V. parahaemolyticus*

**Figure 2:**
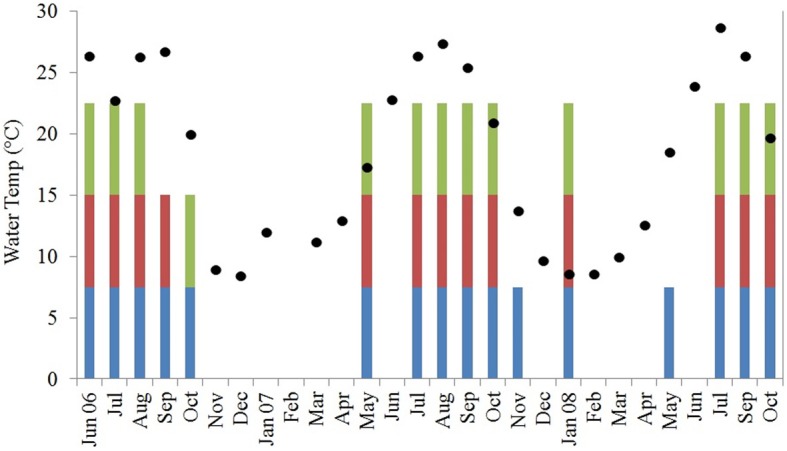
**Water temperature in degrees C (black diamonds, Y-axis) and *V. parahaemolyticus* detection in water (blue bars) and plankton [200 μm (green bars) and 64 μm (magenta bars)]**. Water temperature is averaged across all sites for each sampling month and colored bars demonstrate at least 1 positive sample for that fraction across all sites for each sampling month.

In total, 170 isolates of *V*. *parahaemolyticus* were recovered from Black Sea water and plankton samples collected along the Georgian coast, of which 101 were from water, 30 from the 64 μm fraction, and 39 from the 200 μm fraction of plankton (Figure 2).

*Vibrio parahaemolyticus* was isolated from 40 of a total of 106 water samples collected and 19 of 106 and 26 of 106 of 64- and 200-μm plankton fractions, respectively. Based on Cochran's *Q*-test, water samples yielded *V. parahaemolyticus* significantly more frequently than either of the plankton fractions. The difference in *V. parahaemolyticus* isolation frequency was not significantly different between the two plankton fractions. When these distributions were binned to water temperature quartiles (11, 19.8, and 25.8°C), water samples with temperature between 11 and 19.8°C were significantly more likely to yield *V. parahaemolyticus* isolates than plankton.

Median water temperatures and salinities for all fractions positive for *V. parahaemolyticus* were higher than those that were negative for *V. parahaemolyticus*, while the opposite was observed for dissolved oxygen (Table 1). Median pH levels were slightly lower for all fractions positive for *V. parahaemolyticus* than those that were negative, excluding the P64 fraction (Table 1).

**Table 1:** **Recorded environmental parameters when *V. parahaemolyticus* was/was not detected for each sample type**.

**Environmental conditions when *V. parahaemolyticus* was detected/not detected**					
**Media**	**Statistic**	**Salinity (**‰**)**	**Water temp (°C)**	**pH**	**DO (mg/L)**
Water	Min	3.4 A/3.6 B	8/7.7	6.2/6.3	2.1/2
	Max	20.8/20.8	28.5/29.7	8.6/8.5	7.2/7
	Mean	12.9/12	22.8/16.5	7.7/7.8	4.4/4.3
	Median	15.7/13	24.25/13	7.8/7.9	4.3/4.6
	Std Dev	5.0/7.3	4.9/7.3	0.7/0.6	1.2/1.3
P64	Min	5/3.6	19.3/7.7	6.5/6.2	2/2
	Max	17.4/20.8	28.5/29.7	8.4/8.5	6.8/7.2
	Mean	13.6/12.3	25.4/17.3	7.9/7.8	4.1/4.4
	Median	16.5/14.2	26.6/17	8.2/7.9	4.2/4.5
	Std Dev	4.6/4.9	2.9/7	0.6/0.6	1/1.2
P200	Min	3.4/3.6	18/7.7	6.2/6.5	2.1/2
	Max	20.8/20.8	29/29.7	8.4/8.5	7.2/7.2
	Mean	12.8/12.3	24.6/17	7.6/7.8	4.4/4.3
	Median	14.9/14	25.6/14.2	7.6/8	4.1/4.4
	Std Dev	5.4/4.7	3/7	0.7/0.5	1.3/1.2
All Plankton	Min	3.4/3.6	18/7.7	6.2/6.5	2/2
	Max	20.8/20.8	29/29.7	8.4/8.5	7.2/7.2
	Mean	13.2/12.3	25/16.2	7.7/7.8	4.2/4.3
	Median	16/14.1	25.8/13.6	7.8/8	4.1/4.5
	Std Dev	5/4.8	3/6.8	0.7/0.5	1.2/1.2
All Sample Types	Min	3.4/3.6	8/7.7	6.2/6.2	2/2
	Max	20.8/20.8	29/29.7	8.6/8.5	7.2/7.2
	Mean	12.7/12	22.7/15.3	7.7/7.8	4.4/4.3
	Median	15.2/13.2	24/12.4	7.7/8	4.4/4.6
	Std Dev	5/4.9	4.9/6.9	0.7/0.5	1.2/1.2

*A, statistic when V. parahaemolyticus was detected*.

*B, statistic when V. parahaemolyticus was not detected*.

### Serodiversity

Twenty-seven serotypes of *V. parahaemolyticus* were detected the majority of which were O2:K28 (7 isolates), O3:K31 (7), O3:KUT (7), O4:KUT (7), and untypable (24) (Table 2). *Vibrio parahaemolyticus* O3 O-antigenic type was the most common, comprising 35% of the isolates. Untypable strains may represent strains with novel serology for which *V. parahaemolyticus* anti-sera has not yet been developed, or strains in which antigenic expression is altered or repressed.

**Table 2:** **Molecular characteristics of serotyped strains**.

**Serotype**	**No. of isolates**	***tdh***	**KP**	***trh***	**Mtase**	***ureC***	**VP 1680**	**VP 1686**	**VPA1321 (*vopC*)**	**Vp 1346 (*yop*)**	**VPA 1339 (*escC*)**	**HU-a ORF**	**ORF8**	**GS-PCR**	**VPF2/VPR2**
O1:K32	1	0	0	0	0	0	l^a^ (100^b^)	1 (100)	0	0	0	0	0	0	0
O1:K58	1	0	0	0	0	0	1 (100)	1 (100)	0	0	0	0	0	0	1 (100)
O1:KUT	1	0	0	0	0	0	1 (100)	1 (100)	0	0	0	0	0	1 (100)	1 (100)
O2:K28	7	0	0	0	0	0	7 (100)	7 (100)	0	0	0	0	0	0	0
O2:KUT	1	0	0	0	0	0	1 (100)	1 (100)	0	0	0	0	0	0	0
O3:K5	1	0	0	0	0	0	1 (100)	1 (100)	0	0	0	0	0	0	0
O3:K31	7	0	0	0	0	0	7 (100)	7 (100)	0	0	0	0	0	1 (14)	2 (29)
O3:K33	1	0	0	0	0	0	1 (100)	1 (100)	0	0	0	0	0	1 (100)	1 (100)
O3:K51	2	0	0	0	0	0	2 (100)	2 (100)	0	0	0	0	0	0	0
O3:K65	2	0	0	0	0	0	2 (100)	2 (100)	0	0	0	0	0	1 (50)	0
O3:KUT	7	0	0	0	0	0	7 (100)	7 (100)	0	0	0	0	0	1 (14)	2 (29)
O4:K12	1	0	0	0	0	0	1 (100)	1 (100)	0	0	0	0	0	0	0
O4:K34	1	0	0	0	0	0	1 (100)	1 (100)	0	0	0	0	0	0	0
O4:K37	1	0	0	0	0	0	1 (100)	1 (100)	0	0	0	0	0	0	
O4:KUT	7	0	0	0	0	0	7 (100)	7 (100)	0	0	0	0	0	0	2 (29)
O5:K68	2	0	0	0	0	0	2 (100)	2 (100)	0	0	0	0	0	0	1 (50)
O5:KUT	2	0	0	0	0	0	2 (100)	2 (100)	0	0	0	0	0	0	1 (50)
O6:KUT	1	0	0	0	0	0	1 (100)	1 (100)	0	0	0	0	0	0	0
O8:KUT	2	0	0	0	0	0	2 (100)	2 (100)	0	0	0	0	0	0	0
O10:K61	1	0	0	0	0	0	1 (100)	1 (100)	0	0	0	0	0	0	0
O10:K60	1	0	0	0	0	0	1 (100)	1 (100)	0	0	0	0	0	0	0
O10:KUT	1	0	0	0	0	0	1 (100)	1 (100)	0	0	0	0	0	0	1 (100)
O11:KUT	1	0	0	0	0	0	1 (100)	1 (100)	0	0	0	0	0	0	1 (100)
OUT:K27	1	0	0	0	0	0	1 (100)	1 (100)	0	0	0	0	0	0	0
OUT:K33	2	0	0	0	0	0	2 (100)	2 (100)	0	0	0	0	0	1 (50)	1 (50)
OUT:K52	1	0	0	0	0	0	1 (100)	1 (100)	0	0	0	0	0	0	1 (100)
UT	24	0	0	0	0	0	24 (100)	24 (100)	0	0	0	0	0	1 (4)	12 (50)
Total	80	0	0	0	0	0	80 (100)	80 (100)	0	0	0	0	0	7 (9)	27 (34)

a *Number of positive isolates*.

b *Percent of total isolates of that serotype*.

### Virulence factors, markers of pandemic clones, and rep-PCR

**Figure 3:**
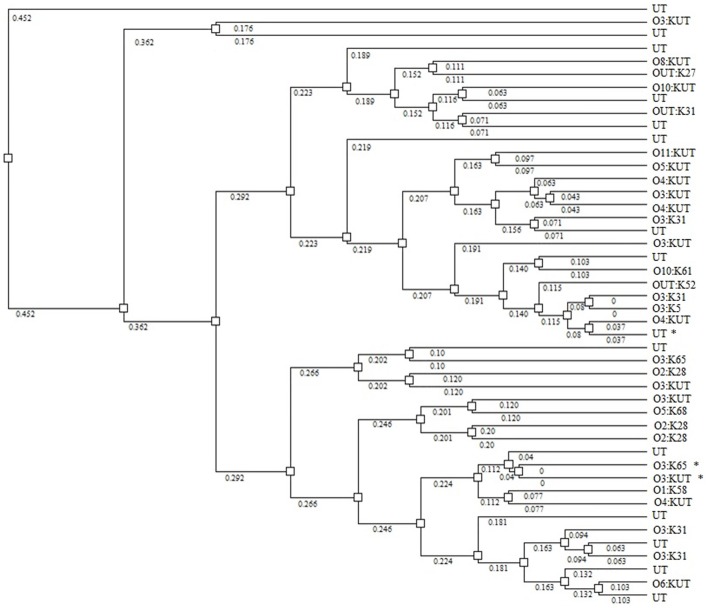
**Dendrogram showing relatedness of *V. parahaemolyticus* strains by rep-PCR**. Asterisks identify strains that are GS-PCR-positive. Numbers on branches indicate degree of divergence between isolates.

None of the *V. parahaemolyticus* isolates carried the genes for thermostable direct hemolysin (*tdh*) thermostable-related hemolysin (*trh*), TTSS-2, or MTase; all were both, Kanagawa phenomenon and urease negative (Table 2). Nineteen isolates resulted in PCR amplicons for the pandemic GS-PCR marker (*toxRS* sequence of pandemic strains), but only seven were 651 bp and 12 were ca. 750 bp. Twenty seven isolates carried the 850-bp pandemic sequence (VPF2/VPR2). Three of the 651 bp, GS-PCR-positive strains were positive for the 850 bp pandemic sequence, whereas six of the 750 bp, GS-PCR-positive isolates encoded this region. Each of the 651 bp, GS-PCR-positive isolates were different serotypes and were typed as O1:KUT, O3:KUT, O3:K31, O3:K33 O3:K65, OUT:K33, and UT, the most notable was the O1:KUT, related to pandemicity. This isolate was also positive for the 850 bp pandemic sequence but lacked all other markers of virulence except TTSS1. Rep-PCR was performed on 45 of the strains (Figure 3). A dendrogram of banding patterns revealed a high level of diversity suggesting a non-clonal population of *V. parahaemolyticus* in this environment.

### Predictive modeling

Among four explanatory variables in a logistic regression used to model presence/absence of *V. parahaemolyticus* as the response variable, water temperature was the only significant predictor (Table 3). When data from all sites were combined, water temperature explained 37.3% of variance in isolation of *V. parahaemolyticus*, suggesting the dynamics of the population are driven by multiple factors. In the Chorokhi and Supsa estuaries, the proportion of variance in *V. parahaemolyticus* isolation explained by water temperature was 22 and 32.1%, respectively, but higher for Batumi Bulvard and Green Cape sites, 43.2 and 70.1%, respectively (Table 3).

**Table 3:** **Results of binary logistic regression analysis between *V. parahaemolyticus* water temperature**.

**Parameter**	**All Sites**	**Chorokhi**	**Batumi Bulvard**	**Green Cape**	**Supsa**
	**Temperature**				
% of Total Variance	37.33	22.01	43.18	70.08	31.23
*P*-value	8.00E-10	0.008	0.004	0.005	0.003
Coefficient	0.27	0.19	0.32	0.53	0.24
Intercept	−5.51	−4.09	−6.79	−9.36	−4.99
Deviance	112.93	35.47	25.98	12.32	30.16
*df*. residual	128	31	31	30	30
*df*. null	129	32	32	31	31
*N*	130	33	33	32	32

## Discussion

Although commonly isolated from brackish waters, presence of *V. parahaemolyticus* suggests a public health concern to those utilizing these water sources or consuming products harvested from these waters. This risk is appreciable regardless of pathogenicity island presence in the genomes of circulating *V. parahaemolyticus*, since some infections are caused by isolates lacking *tdh*, *trh*, and TTSS2 (Suthienkul et al., 1995; Okuda et al., 1997; Vuddhakul et al., 2000; Laohaprertthisan et al., 2003; Cabanillas-Beltrán et al., 2006; Bhoopong et al., 2007; Meador et al., 2007; Serichantalergs et al., 2007; Chao et al., 2009, 2010; García et al., 2009; Harth et al., 2009). Isolates recovered in this study lacked the major virulence factors associated with the majority of clinical cases. However, these results are not surprising since typically <1% of environmental isolates encode these elements (McLaughlin et al., 2005). The historical reporting of *V. parahaemolyticus* infections in this region suggests that either infections have been caused by strains lacking major virulence factors, resident strains encoding these virulence factors were not detected using the methods employed by this study, or both.

Results of this study demonstrated a high level of diversity among isolates as measured by serotype distribution, presence/absence of pandemic markers, and rep-PCR banding patterns. Strains isolated in this study represented 9 O-antigens and 27 K-antigens, as well as untypable strains, a measure of antigenic diversity of natural isolates in this region. Mutations within antigen coding regions of the genome are common, as well as lateral transfer, allowing strains to adapt to microenvironments of the environment or evade predation by grazing protozoa (Lerouge et al., 2001; Woo et al., 2001; Wildschutte et al., 2004). Molecular divergence was noted by the heterogeneity observed among O3:K31 and O2:K28 strains by rep-PCR analysis suggesting that serology does not necessarily correlate with genome architecture. This genomic heterogeneity indicates the necessity of classifying strains by methods other than serology. The high degree of divergence among environmental *V*. *parahaemolyticus* strains in the Black Sea is corroborated by reports of similar findings in geographically distant regions (Wong et al., 1999; Matsumoto et al., 2000; Alam et al., 2009; Yu et al., 2002; Ellis et al., 2012; Paranjpye et al., 2012).

*V. parahaemolyticus* was detected across a broad range of salinities (3.4–20.8‰) (Table 2). However, it was not significantly associated with *V. parahaemolyticus* presence in our model. This is most likely due to the relative stability of salinity readings at each site over the course of the study (data not shown). *V. parahaemolyticus* is a known member of estuarine and marine environments and salinity values detected during this study were typical of brackish waters (0.5 > 30‰) suggesting a suitable salinity regime for *V. parahaemolyticus* presence at most sampling points. *V. parahaemolyticus* seasonality was observed at all sites, with a clear trend of increasing numbers as water temperatures increased from May to September. The organism was isolated from water samples at temperatures as low as 8°C, but more frequently (ca. 93% of strains) at temperatures greater than 17°C (Table 1). The highest percentage of total variance in detection, related to temperature, was at Green Cape (percent of total variance = 70, *P* < 0.05). At each site, the total variance in *V. parahaemolyticus* detection was significantly related to an increase in water temperature. However, these associations were not as strong for the Batumi Bulvard (43.18), Chorokhi estuary (22.01), and Supsa estuary (31.23) sites (Table 3). Interestingly, the associations between water temperature and *V. parahaemolyticus* detection were weaker for the two estuarine sites. Salinities at these two sites were much lower than the non-estuarine sites (Batumi and Green Cape) suggesting that either salinity played a role in *V. parahaemolyticus* presence, even though it did not show up as significant in our model, or that an unmonitored parameter common to both estuarine environments influenced *V. parahaemolyticus* presence. This trend is indicative of the patchiness of *V. parahaemolyticus* distribution in water bodies suggesting that environmental conditions are noticeably different at different locations within the same water body and that these differences contribute to *V. parahaemolyticus* presence.

In summary, an antigenically diverse population of *V*. *parahaemolyticus* inhabits the Georgian coast of the Black Sea. Although none of the strains collected during this study were Kanagawa phenomena-positive or *tdh* and *trh*-positive, the TTSS1 effector proteins and TLH were present in some isolates, which included a possible serovariant of the *V. parahaemolyticus* O3:K6 pandemic clone. These results, together with epidemiological data demonstrating strains lacking pathogenicity islands can cause disease, suggest there is a risk associated with occurrence of *V*. *parahaemolyticus* in Black Sea coastal waters. Warmer temperatures in the spring and summer lead to increased densities of *V. parahaemolyticus*. Recent clinical data on isolation of TDH-, TRH-, and TTSS2-negative *V*. *parahaemolyticus* suggests these strains represent underreported etiological agents of diarrhea, similar to *V*. *cholerae* non-O1/non-O139 strains lacking major virulence factors (Safrin et al., 1988; Ko et al., 1998; Lukinmaa et al., 2006; Shannon and Kimbrough, 2006; Chatterjee et al., 2009; Hasan et al., 2012; Marin et al., 2013). The high frequency of detection of *V*. *parahaemolyticus* lacking major virulence factors but associated with severe infection, suggests recreational water and shellfish harvesting areas in Georgia should be monitored, especially when water temperatures are seasonally high.

### Conflict of interest statement

The authors declare that the research was conducted in the absence of any commercial or financial relationships that could be construed as a potential conflict of interest. The Associate Editor declares that despite being affiliated to the same institution as the authors Bradd J. Haley, Christopher J. Grim, Arlene J. Chen, Elisa Taviani, Rita R. Colwell and Anwar Huq, the review process was handled objectively and no conflict of interest exists.
